# Supplementary material for: Rapid endogenic rock recycling in magmatic arcs
Source: Nat Commun. 2021 Jun 10;12:3533. doi: 10.1038/s41467-021-23797-3 (PMC8192928; doi:10.1038/s41467-021-23797-3)
Supplement: Supplementary file 1 — Supplementary Information [file 41467_2021_23797_MOESM1_ESM.pdf]

Supplementary Information for

Rapid endogenic rock recycling in magmatic arcs

Jun-Yong Li[1,2], Ming Tang[2,3], Cin-Ty A. Lee[2], Xiao-Lei Wang[1]*, Zhi-Dong Gu[4], Xiao-Ping Xia[5], Di Wang[1], De-Hong Du[1] & Lin-Sen Li[1]

[1]State Key laboratory for Mineral Deposits Research, School of Earth Sciences and Engineering, Nanjing University, Nanjing 210023, China
[2]Department of Earth, Environmental and Planetary Sciences, Rice University, Houston, Texas 77005, USA
[3]School of Earth and Space Sciences, Peking University, Beijing 100871, China
[4]Research Institute of Petroleum Exploration and Development, PetroChina, Beijing 10083, China
[5]State Key Laboratory of Isotope Geochemistry, Guangzhou Institute of Geochemistry, Chinese Academy of Sciences, Guangzhou, 510640, China

*Corresponding author: Xiaolei Wang (wxl@nju.edu.cn)

Supplementary Figure 1

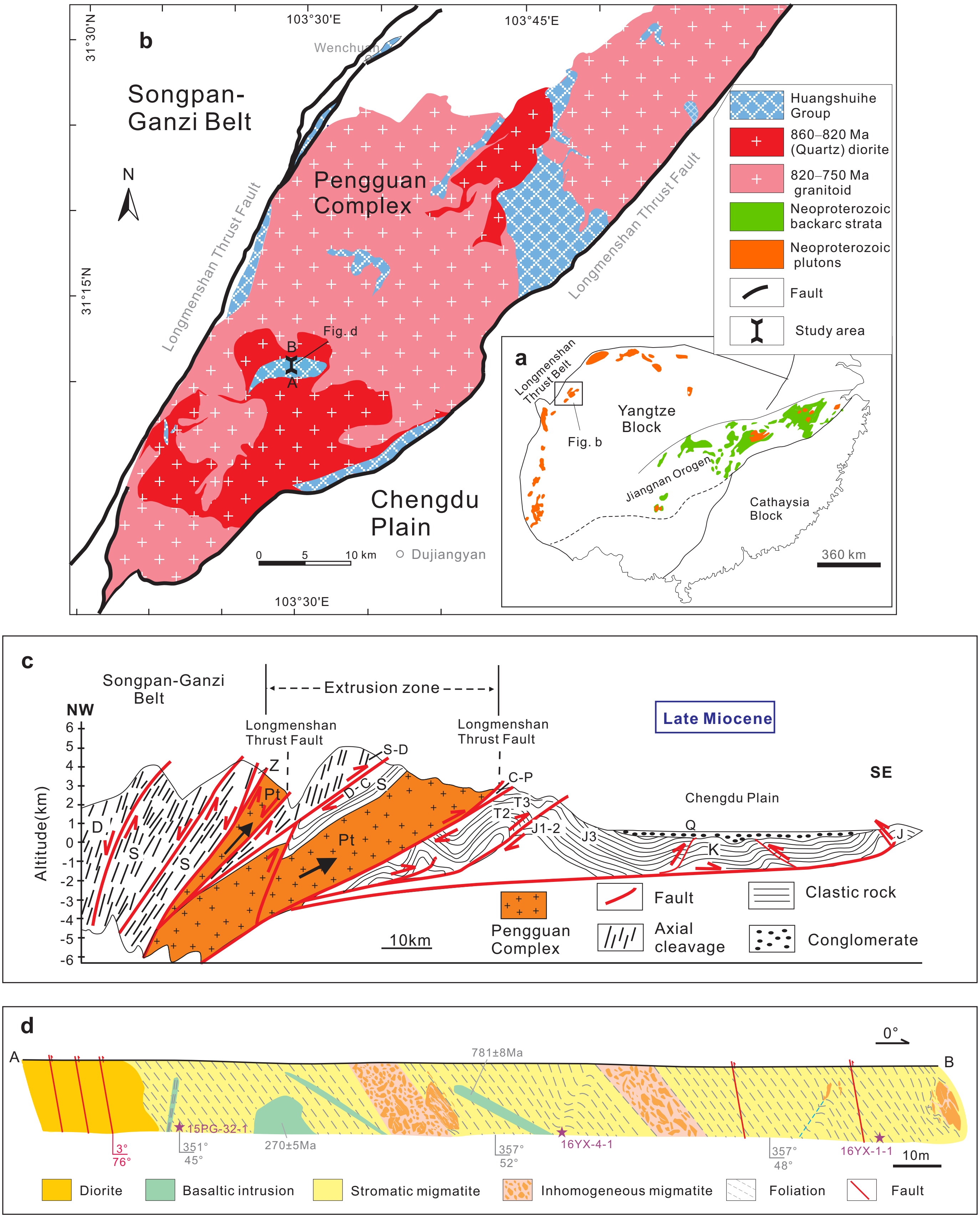

Supplementary Figure 1: Geological sketch map of study area. a Simplified geologic map showing linearly distributed Neoproterozoic plutonic rocks (orange color) along western Yangtze Block; Jiangnan orogen outcrops extensive, thick Neoproterozoic back-arc strata (green

color). **b** Zoom-in map showing rock configuration of the Pengguan Complex and studied location; 860–820 Ma (quartz) diorite in Pengguan Complex correlated with continental arc magmatism while the protoliths of migmatites (this study) in the Huangshuihe Group formed between 860–820 Ma. **c** Cross section of the Longmenshan thrust belt showing the uplift of the Pengguan Complex resulted from major Miocene extrusion (modified after Wang and Meng, 2009). **d** Cross-section illustrating stromatic and inhomogeneous migmatites, mafic dikes and diorite intrusion in study area.

Supplementary Figure 2

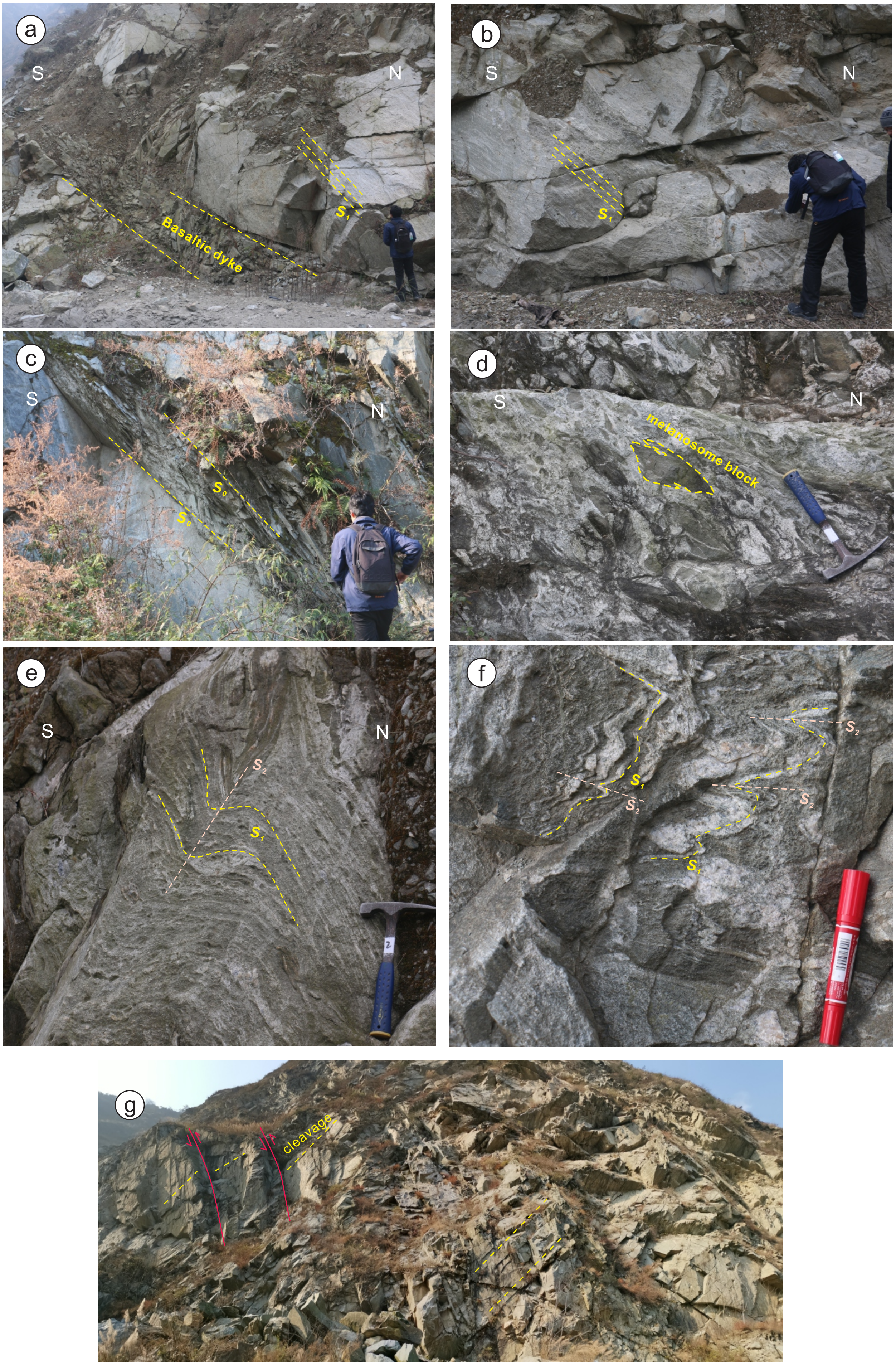

Supplementary Figure 2: Field photos showing two different types of migmatite, patch-shaped neosome, layering structure, foliation, and syn-anatexis deformation structure. **a&b** Typical stromatic migmatites with melts generated along the foliation planes (S_1 mean: 355°/48°). **c** The metapelite in the study area shows clear sedimentary bedding structure (S_0: 331°/61°), with its orientation nearly parallel to the S_1 foliation in stromatic migmatite. **d** The inhomogeneous migmatite containing abundant blocks of melanosome and associated apline vein. **e&f** The S_1 foliation is locally folded by syn-anatectic deformation and the fold axial plane (S_2) is generally E–W striking and S-dipping. **g** Massive high-angle thrust faults observed in the study area. They should be associated with post-Mesozoic extrusion process which led to the exposure of the Pengguan Complex.

Supplementary Figure 3

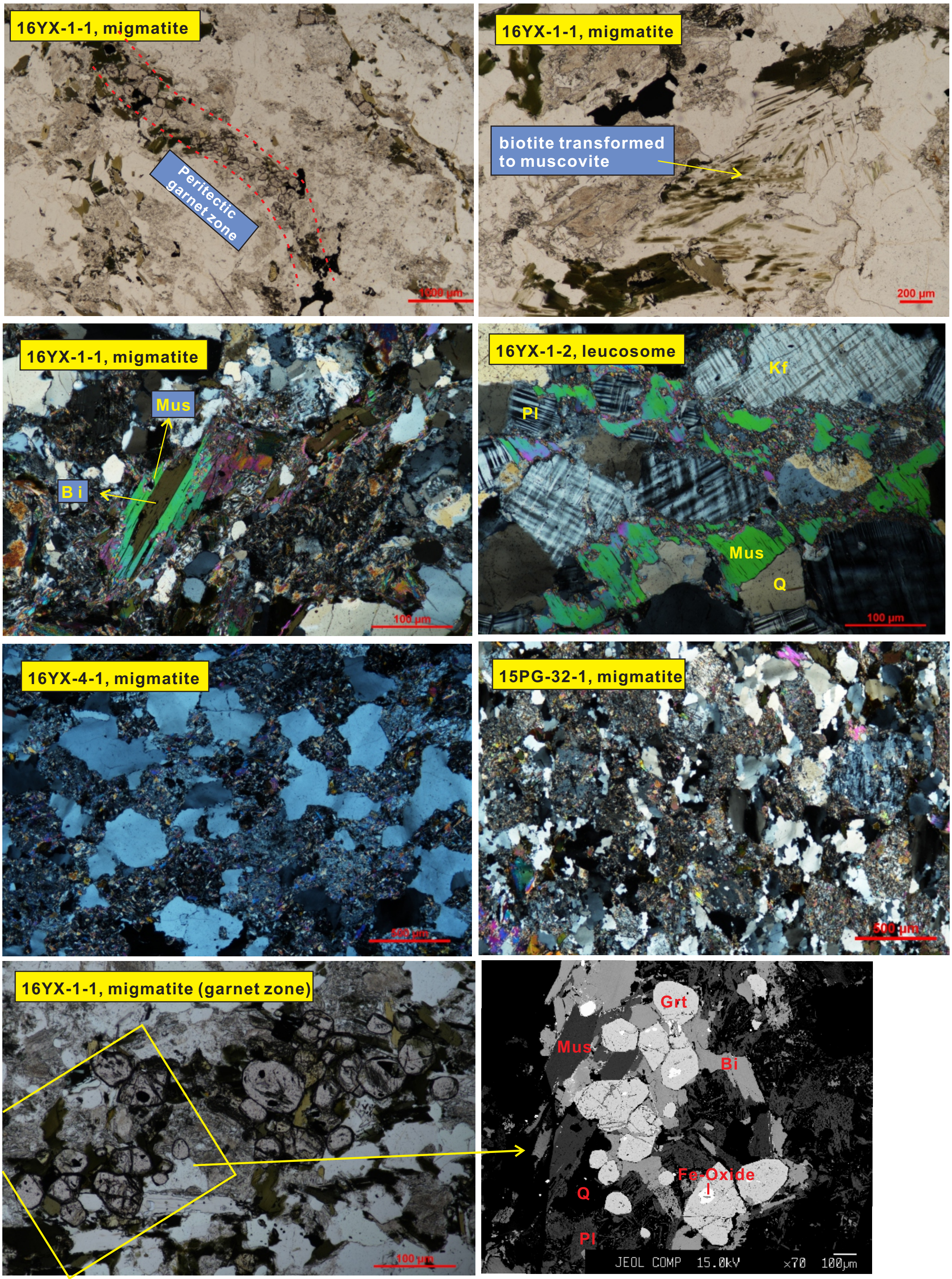

Supplementary Figure 3: Selected photomicrographs and back scattered electron (BSE) images showing mineral structure, composition and metamorphic reaction in the migmatite and leucosome samples. Grt–garnet, Bi–biotite, Mus–muscovite, Pl–plagioclase, Kf–K-feldspar, Q–quartz.

Supplementary Figure 4

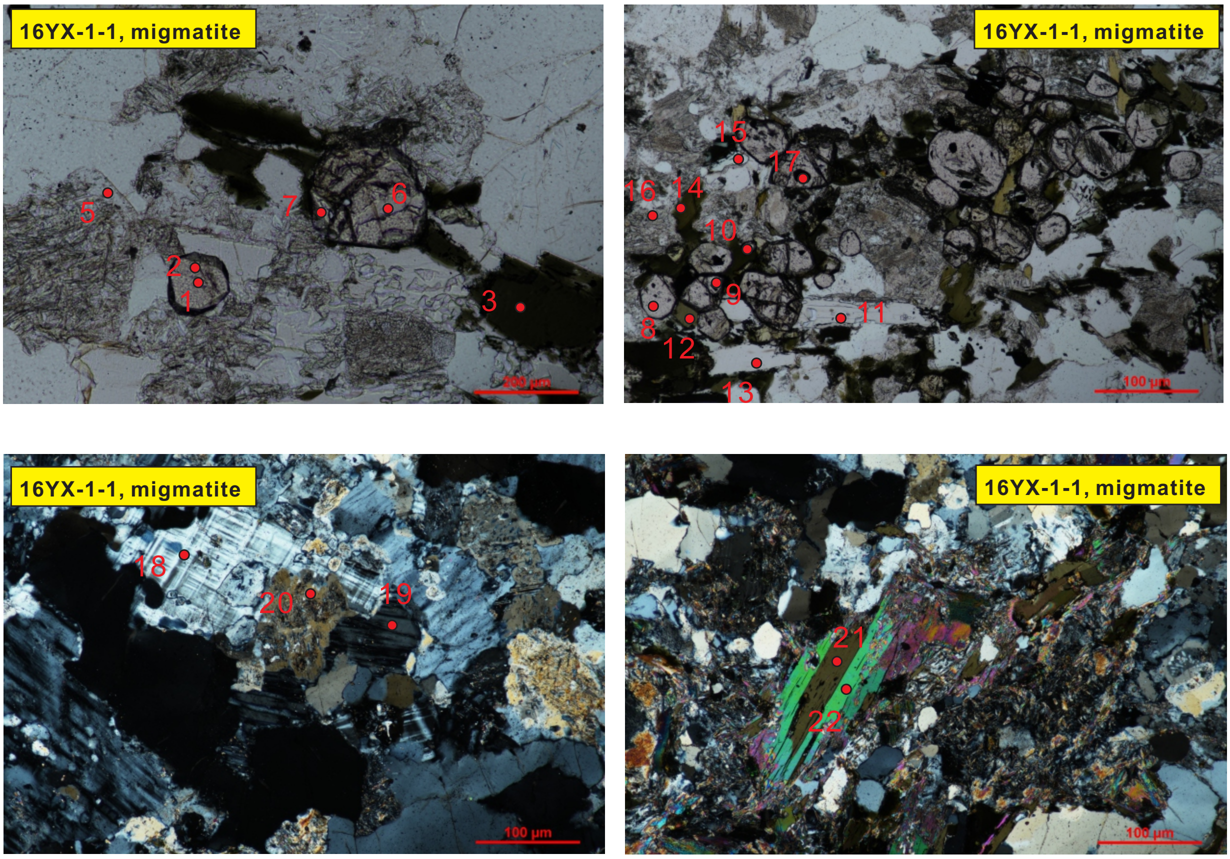

Supplementary Figure 4: Selected analytical spots for mineral composition. Results are shown in Supplementary Data 4.

Supplementary Figure 5

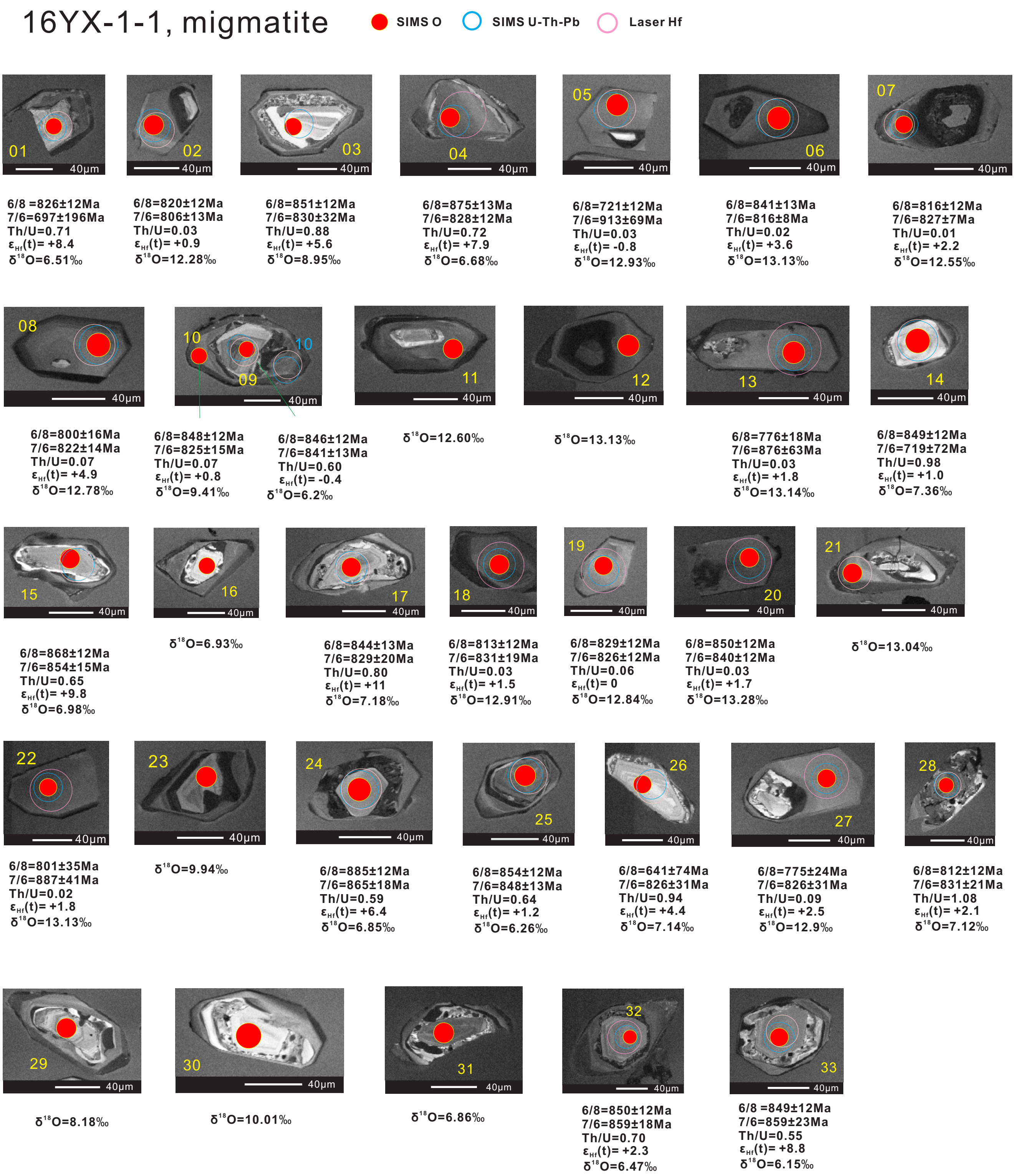

Supplementary Figure 5: CL images of dated rim and core zircon from the sample 16YX-1-1, with dated results of $^{206}Pb/^{207}Pb$ age (refer to 7/6 in figure), $^{206}Pb/^{238}U$ age (refer to 6/8 in figure), Th/U ratio, $\varepsilon_{Hf}(t)$ and $\delta^{18}O$ values marked. Detailed data could be found in Supplementary Data 3.

Supplementary Figure 6

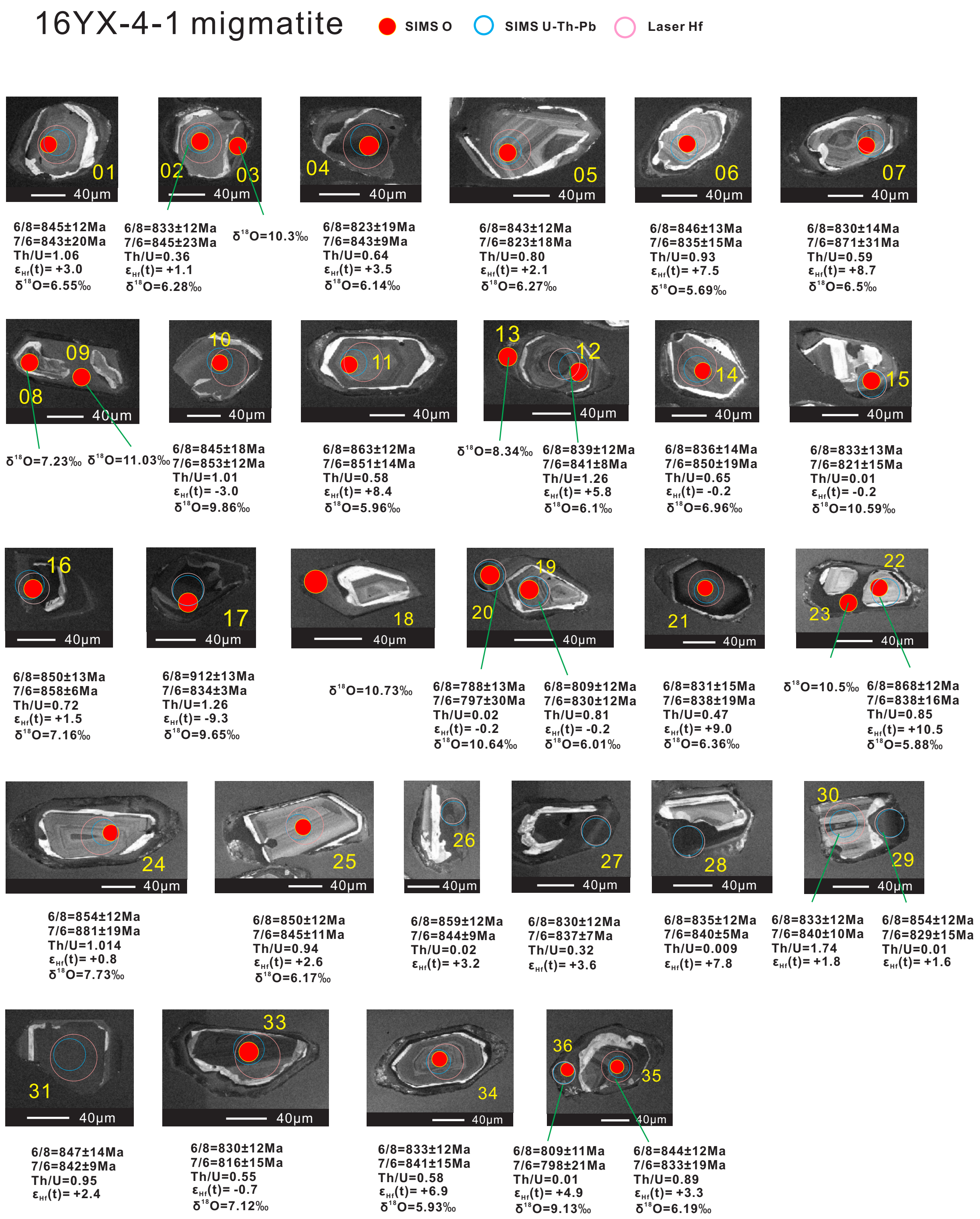

Supplementary Figure 6: CL images of dated rim and core zircon from the sample 16YX-4-1, with dated results of $^{206}Pb/^{207}Pb$ age (refer to 7/6 in figure), $^{206}Pb/^{238}U$ age (refer to 6/8 in figure), Th/U ratio, $\varepsilon_{Hf}(t)$ and $\delta^{18}O$ values marked. Detailed data could be found in Supplementary Data 3.

Supplementary Figure 7

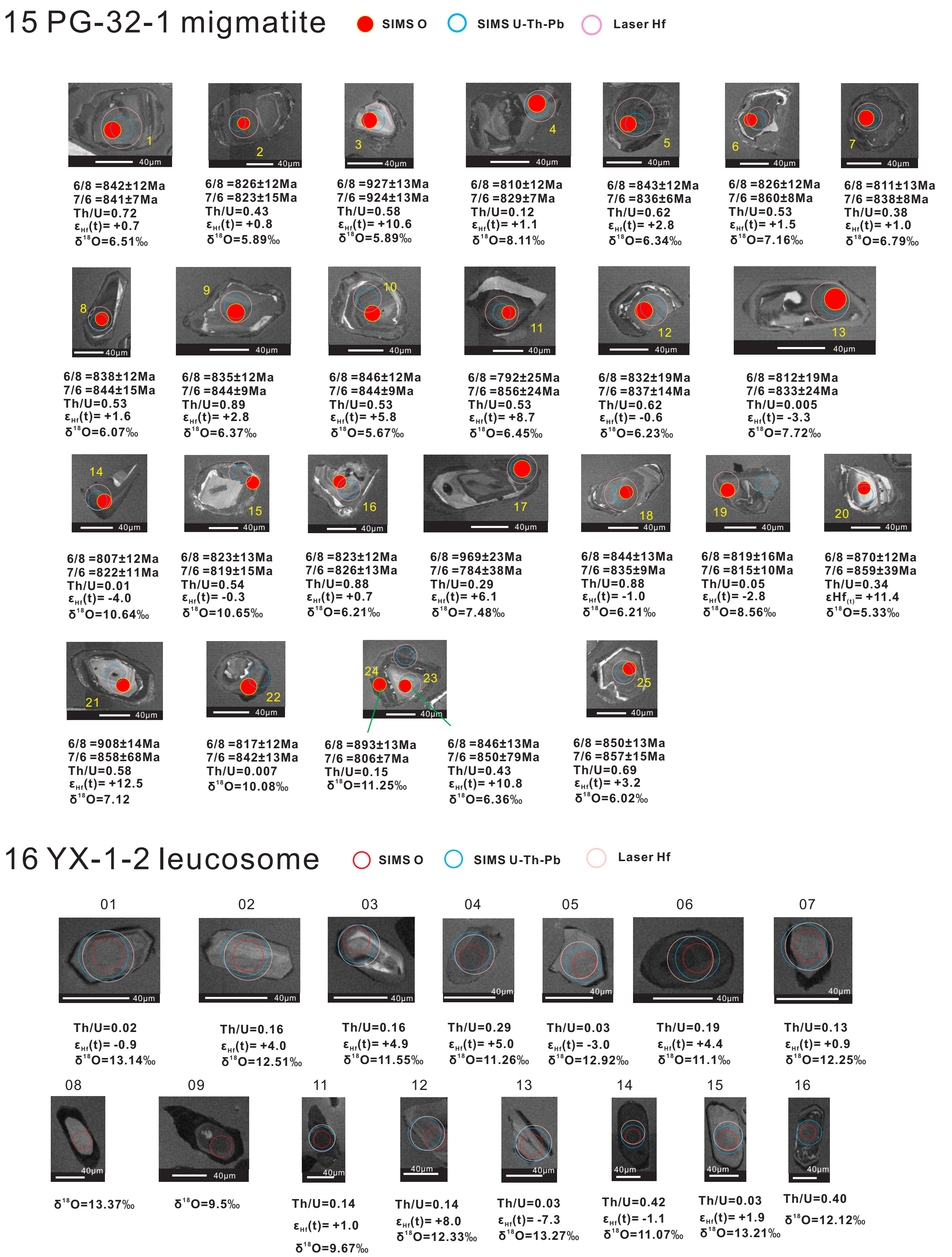

Supplementary Figure 7: CL images of dated rim and core zircon from the samples 15PG-32-1 and 16YX-1-2, with dated results of $^{206}Pb/^{207}Pb$ age (refer to 7/6 in figure), $^{206}Pb/^{238}U$ age (refer to 6/8 in figure), Th/U ratio, $\varepsilon_{Hf}(t)$ and $\delta^{18}O$ values marked. Detailed data could be found in Supplementary Data 3.

Supplementary Figure 8

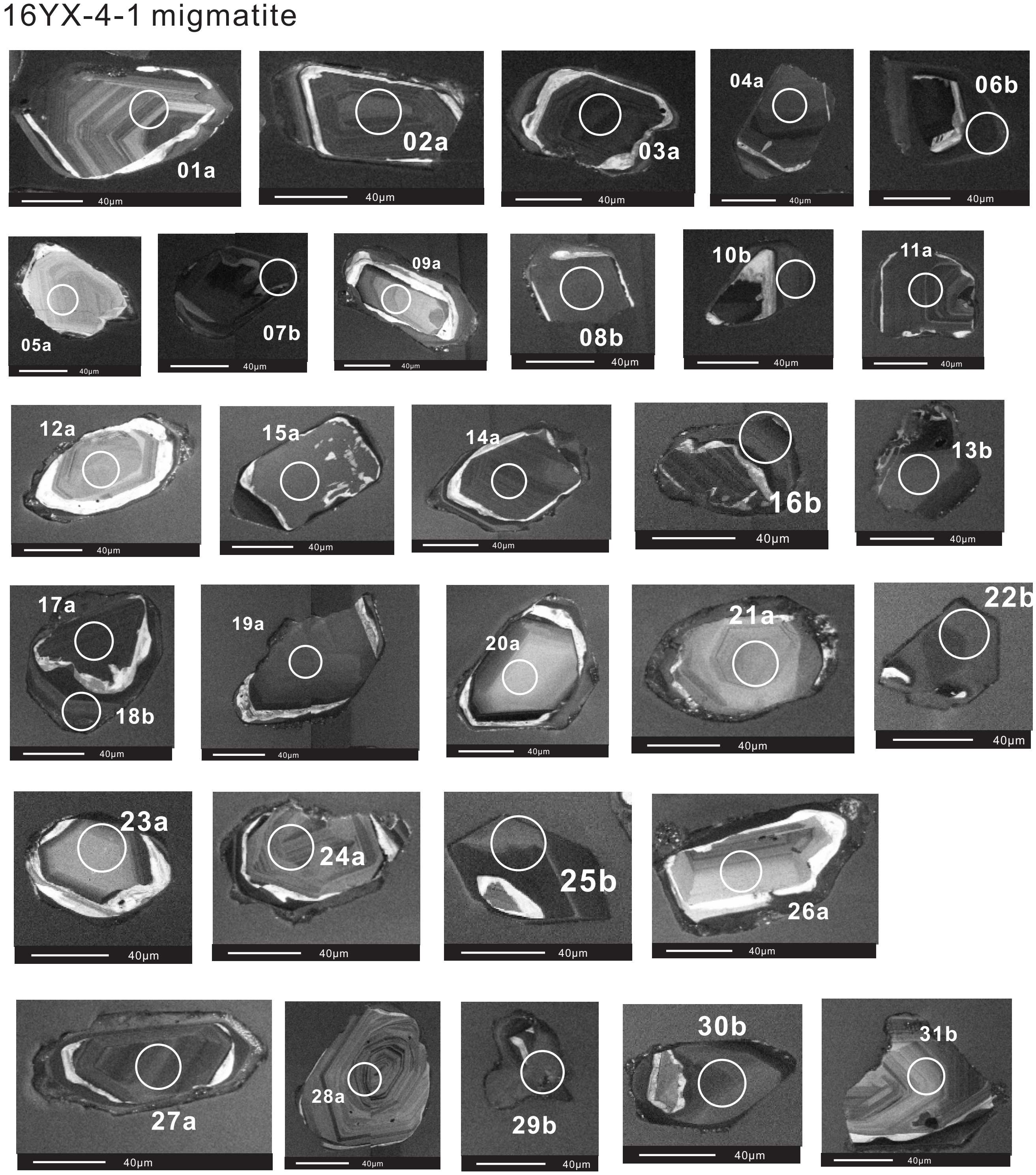

Supplementary Figure 8: Selected analytical spots from sample 16YX-4-1 for zircon trace element. Results are shown in Supplementary Data 5.

Supplementary Figure 9

16YX-1-1, migmatite

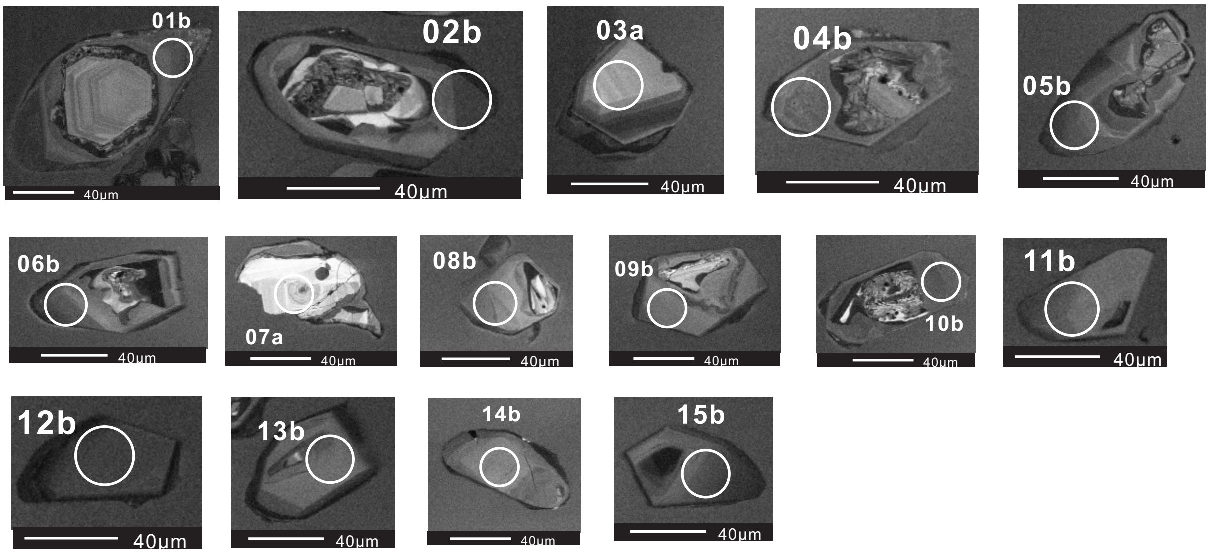

15PG-32-1, migmatite

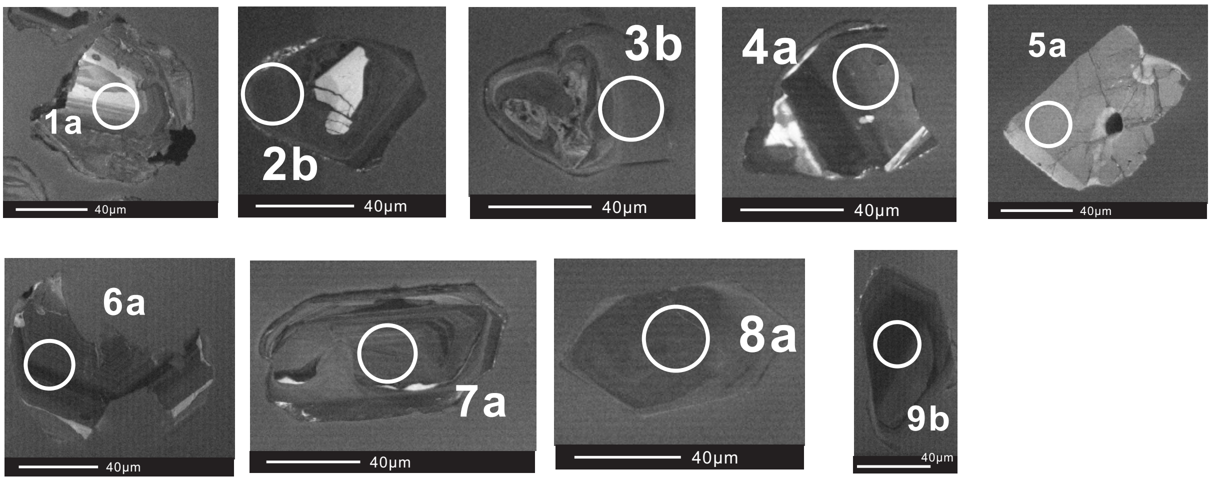

Supplementary Figure 9: Selected analytical spots from samples 16YX-1-1 and 15PG-32-1 for zircon trace element. Results are shown in Supplementary Data 5.
