# Supplementary material for: Rapid endogenic rock recycling in magmatic arcs
Source: Nat Commun. 2021 Jun 10;12:3533. doi: 10.1038/s41467-021-23797-3 (PMC8192928; doi:10.1038/s41467-021-23797-3)
Supplement: Supplementary file 3 — Description of Additional Supplementary Files [file 41467_2021_23797_MOESM3_ESM.pdf]

Description of Additional Supplementary Files

File name: Supplementary Data 1
Description: Major (wt %) and trace element (ppm) compositions of the migmatite and leucosome samples

File name: Supplementary Data 2
Description: Summary of Age- δ18O -εHf(t) results from core and rim zircon of the migmatites

File name: Supplementary Data 3
Description: Analytical results of zircon U-Pb-Hf-O dating for migmatite and leucosome samples in the Huangshuihe Group

File name: Supplementary Data 4
Description: Major element (wt. %) results for garnet, muscovite, biotite, plagioclase, K-feldspar and Fe-oxide in 16YX-1-1

File name: Supplementary Data 5
Description: Trace element compositions of zircon grains from the migmatite samples in Huangshuihe Group (ppm)
